# P-1635. Impact of Pre-operative COVID-19 Diagnoses on Post-operative Outcomes in Laparoscopic Cholecystectomy: A Matched Cohort Analysis

**DOI:** 10.1093/ofid/ofaf695.1811

**Published:** 2026-01-11

**Authors:** Meghana Nair, Victoria Zhao, Musa Salaam

**Affiliations:** Quinnipiac University Frank H. Netter School of Medicine, Hamden, CT; Quinnipiac University Frank H. Netter School of Medicine, Hamden, CT; Quinnipiac University Frank H. Netter School of Medicine, Hamden, CT

## Abstract

**Background:**

The consequences of a COVID-19 diagnosis continue to impact patient outcomes beyond respiratory-related procedures. The implications of a COVID-19 diagnosis for laparoscopic cholecystectomy complications and post-operative outcomes remains less understood. This study investigates the impact a COVID-19 diagnosis has on patients’ hospitalization duration, time to surgery, and post-operative outcomes in laparoscopic cholecystectomy.

**Methods:**

The NSQIP database was used to analyze data from laparoscopic cholecystectomy procedures performed in participating hospitals between 2021-2022. A 1:2 propensity score matched group analysis was conducted to evaluate differences in post-operative complication rates, time to surgery, total operation time, and hospital length of stay. Patients were categorized into two cohorts: those with pre-operative COVID-19 and those without. Propensity scores were generated using logistic regression and modeled using demographic and pre-operative clinical features. Outcomes were assessed using GGE marginal models.

**Results:**

A total of 2,583 patients who underwent laparoscopic cholecystectomy were analyzed, with 1,722 in the PreOp COVID-negative group and 861 in the positive group. Patients with pre-operative COVID-19 had a higher risk of post-operative complications (OR = 2.01, 95% CI: 1.45–2.77, p < 0.001), with 8% experiencing complications compared to 4% in the non-COVID group. Time from admission to surgery was prolonged among PreOp COVID patients (1.61 days, 95%CI: 1.46-1.78 vs. 0.76 days, 95%CI: 0.70-0.84, p < 0.001). Additionally, they had longer hospital stays (3.02 days, 95%CI: 2.77-3.28 vs. 1.53 days, 95%CI: 1.41-1.66, p < 0.001). PreOp COVID patients also required longer operative times (78.7 min, 95%CI:75.8-81.6 vs. 68.89 min, 95%CI: 67.1-70.7, p < 0.001).

**Conclusion:**

Pre-operative COVID-19 was associated with increased post-operative complications, increased time to surgery, longer operative times, and prolonged hospital stays. These findings suggest that pre-operative COVID-19 may impact surgical readiness and recovery, potentially leading to delays in care and adverse outcomes. Enhanced pre-operative screening and precautions may be warranted for patients with recent COVID-19 infections to improve outcomes.

**Disclosures:**

All Authors: No reported disclosures

